# Association between uric acid to albumin ratio and all-cause mortality among United States adults: An observational study

**DOI:** 10.1097/MD.0000000000046123

**Published:** 2025-11-21

**Authors:** Suling Ye, Jie Li, Liang Chen, Mengmeng Shan, Jinzhou Zhu, Lili Wang

**Affiliations:** a Department of Cardiology, Ruijin-Hainan Hospital Shanghai Jiao Tong University School of Medicine (Hainan Boao Research Hospital), Qionghai, China; b Department of Cardiology, The Second Affiliated Hospital of Dalian Medical University, Dalian, China.

**Keywords:** all-cause mortality, cross-sectional study, NHANES, United States adults, uric acid to albumin ratio

## Abstract

This study aimed to investigate if the ratio of uric acid to albumin (UAR) correlates with all-cause mortality. Smooth curve fitting was employed to analyze the association between UAR and all-cause mortality; a threshold saturation effect model was utilized to identify the inflection point of UAR’s impact on all-cause mortality. Furthermore, the association between UAR and all-cause mortality from heart disease was assessed using a competing risks model. This study included 25,437 male participants and 27,090 female participants. Smooth curves fitting revealed a U-shaped relationship, demonstrating a distinct inflection point for UAR and all-cause mortality, with the inflection point for females appearing earlier than for males. Kaplan-Meier curves indicated a significantly elevated risk of death in the high UAR group (1.49 < UAR < 4.65) compared to the low UAR group (0 < UAR < 1.03). UAR emerges as a robust independent prognostic indicator for all-cause mortality and could serve as a readily accessible and valuable novel inflammatory marker for identifying high-risk patients.

## 1. Introduction

Uric acid, the end-product of purine metabolism, is widely recognized as an independent risk factor for various health conditions, including chronic kidney disease, cardiovascular risk, hypertension, diabetes mellitus, metabolic syndrome, and cognitive decline.^[1–6]^ Conversely, albumin, the predominant protein in blood, plays crucial roles such as binding and transporting drugs and substances, maintaining blood oncotic pressure, and influencing circulatory system function.^[7]^ Recent evidence indicates an inverse relationship between serum albumin and conditions like acute kidney injury, venous thromboembolism, and cardiovascular and cerebrovascular diseases.^[8–11]^ Elevated uric acid levels and decreased serum albumin levels have been identified as independent risk factors for various diseases, with predictive value.

Uric acid to albumin ratio (UAR) has emerged as a marker for predicting events related to coronary artery disease (CAD). This marker has been assessed in conditions associated with acute atherosclerotic burden, including endothelial injury, inflammatory response, platelet activation, and increased oxidative stress, such as non-ST-elevation myocardial infarction (NSTEMI) and ST-elevated myocardial infarction (STEMI).^[12,13]^ However, limited studies have explored the relationship between UAR and all-cause mortality. Therefore, we conducted a cross-sectional study from the 1999–2018 National Health and Nutrition Examination Survey (NHANES) database to assess the association between UAR and all-cause mortality in adults.

## 2. Methods

### 2.1. Participants

This study utilized data collected from the NHANES spanning the years 1999 to 2018, focusing on uric acid and albumin levels. The NHANES database comprises regularly updated collections of nationally representative samples conducted by the Centers for Disease Control and Prevention, aimed at assessing the health and nutritional status of individuals of all ages residing in the United States.

**Figure 1.**
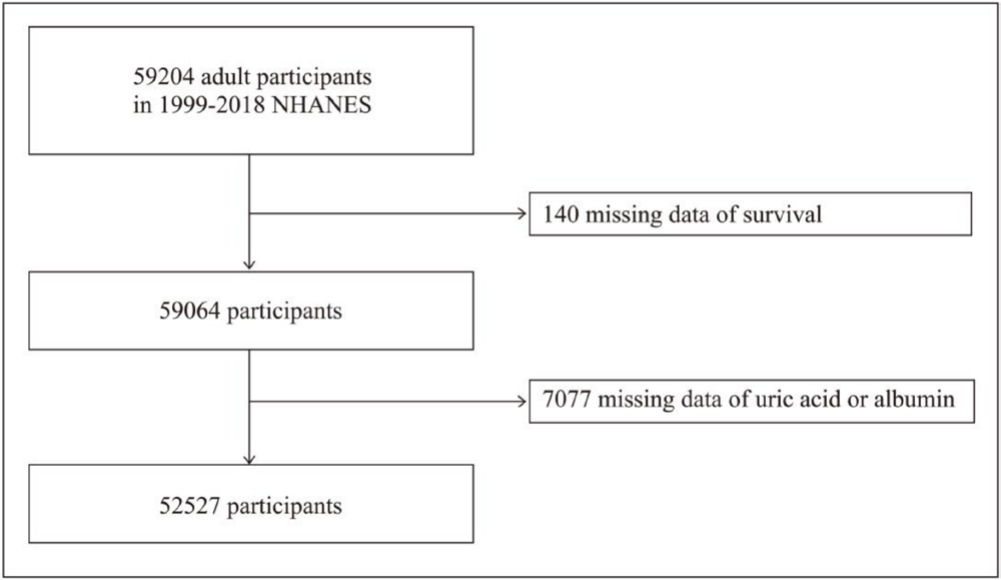
Inclusion and exclusion criteria for study participants. NHANES = National Health and Nutrition Examination Survey.

Out of the 59,204 initially identified participants, 7217 individuals were excluded from the study for various reasons. Specifically, 140 lacked survival status follow-up outcome data, and 7017 lacked uric acid or albumin data. Consequently, only 52,527 eligible adult subjects were included in this study (NHANES 1999–2018). The selection process of participants is illustrated in Figure 1.

### 2.2. Ethical statement

The study protocol received approval from the Ethics Review Committee of the National Center for Health Statistics (NCHS) in the United States, and all participant provided informed consent. All experiments involved in this paper are performed in accordance with relevant guidelines and regulations.

### 2.3. Survival status and follow-up time

NCHS, a government entity, integrates data from various surveys with death certificate records obtained from the National Death Index to explore the correlation between different health factors and mortality. However, in order to safeguard patient privacy, mortality-related variables are only accessible to a limited extent for adult participants, and data perturbation techniques have been employed to mitigate the risk of participant reidentification. For deceased patients, the duration of follow-up since the interview was computed based on the quarter and year of death. For surviving patients, the follow-up duration was calculated from the conclusion of the follow-up period after the death. All data used in this study are sourced from publicly available documents provided by the NCHS, accessible on their website (https://wwwn.cdc.gov/nchs/nhanes/Default.aspx).

### 2.4. Statistics of baseline data

Baseline participant data were collected through a computer-assisted personal interview system, questionnaire surveys, and laboratory tests. The collected data included gender, age, race, and education level (categorized as less than high school, high school/equivalent, and more than high school), family income-to-poverty ratio, and ethnicity (Mexican American, other Hispanics, non-Hispanic White, non-Hispanic Black, other ethnicities), smoking status, alcohol use, presence of diabetes, hypertension, hypercholesterolemia, heart disease, stroke, and body mass index (BMI). Participants were classified as smokers if they had smoked at least 100 cigarettes in their lifetime, while individuals defined as alcohol drinkers had consumed at least 12 alcoholic beverages in the past 12 months. Diseases such as hypertension, hypercholesterolemia, diabetes, stroke, and heart disease (including congestive heart failure (HF), coronary heart disease, angina, or heart attack) were considered diagnosed conditions. BMI was calculated by dividing weight in kilograms by height in meters squared (kg/m^2^). Serum testing was conducted to obtain values for alanine aminotransferase (ALT), aspartate aminotransferase (AST), total bilirubin, total protein, globulin, albumin, creatinine, and uric acid.

### 2.5. Calculation of UAR

UAR was calculated by dividing serum uric acid concentration by serum albumin concentration and was treated as a continuous variable. To mitigate the impact of outliers on the results, participants were stratified into quartiles based on the distribution of participants across UAR values: Q1 (0–1.03), Q2 (1.03–1.24), Q3 (1.24–1.49), and Q4 (1.49–4.65).

### 2.6. Statistical analysis

Missing values in continuous variables, which accounted for <1% of the data, were imputed with the mean of existing data. Categorical variables are presented as percentages. Continuous variables were determined to be non-normally distributed (assessed via the Kolmogorov–Smirnov test). Continuous variables that conformed to the normal distribution are here given as the mean with standard deviation (SD), and those that did not conform to the normal distribution are here given as the median (first quartile and third quartile), while categorical variables are summarized as numbers with percentages. To minimize skewness from extreme values, participants were stratified into quartiles based on the empirical distribution of UAR values across the cohort. The comparison in the different groups was conducted using the chi-square test for categorical variables, Student *t* test for continuous variables that conformed to the normal distribution, and the rank sum test for continuous variables that did not conform to the normal distribution. Spline smoothing plot analysis based on restricted cubic spline functions was employed to examine potential nonlinear associations between the UAR and all-cause mortality risk.^[14]^ The inflection point of UAR’s effect on all-cause mortality was subsequently identified through a threshold saturation effect model based on Cox regression equations, with statistical significance confirmed by log-likelihood ratio tests.^[15]^ Additionally, Kaplan–Meier curves assessed death trends across groups. To quantify the specific association between the UAR and heart disease-related mortality while accounting for competing risks from other causes, a competing risks regression model was employed.^[16]^ Prior to regression analyses, all variables were examined for collinearity, and none exhibited a variance inflation factor exceeding 5. A multivariate Cox regression model was applied to mitigate the influence of confounding factors on the results. Covariates in the model were selected based on the principle of screening for confounding factors: the covariates that changed estimates of the effect of UAR on mortality by more than 10% were adjusted (including age, race, education level, ratio of family income to poverty, BMI, smoking, alcohol use, ALT, AST, total protein, globulin, creatinine, and histories of hypertension, hypercholesterolemia, diabetes, heart disease, and stroke).

The data analyses were carried out using R (http://www.r-project.org; version 3.4.3) and EmpowerStatsXYS 5.2 (https://www.empowerstats.net/cn/). Statistical significance was defined as a *P* value of <.05.

## 3. Results

### 3.1. Baseline characteristics of the participants

The study population’s general characteristics are summarized. Compared with the low UAR group (0 < UAR < 1.03), the high UAR group (1.49 < UAR < 4.65) exhibited higher mortality, with participants being older and having higher levels of ALT, AST, globulin, creatinine, and uric acid, alongside lower albumin levels. Significant differences were also observed in gender, race, education level, household income-to-poverty ratio, BMI, smoking status, alcohol consumption, hypertension, hypercholesterolemia, diabetes, heart disease, and history of stroke (Table 1).

**Table 1:** Baseline characteristics of participants (N = 52,527).

Number (%)/mean ± SD/median (Q1, Q3)	Uric acid to albumin ratio				*P*
	Q1 (0–1.03)	Q2 (1.03–1.24)	Q3 (1.24–1.49)	Q4 (1.49–4.65)	
13,115	13,130	13,102	13,180
Death					<.001
No	11,878 (90.6)	11,575 (88.2)	11,294 (86.2)	10,157 (77.1)	
Yes	1237 (9.4)	1555 (11.8)	1808 (13.8)	3023 (22.9)	
Time, mo	121.0 (68.0–187.0)	116.0 (64.0–175.0)	113.0 (61.0–170.0)	100.0 (50.0–155.2)	<.001
Age, yr	40.0 (27.0–56.0)	45.0 (29.0–61.0)	48.0 (31.0–64.0)	55.0 (38.0–69.0)	<.001
Sex					<.001
Male	2811 (21.4)	5794 (44.1)	7851 (59.9)	8981 (68.1)	
Female	10,304 (78.6)	7336 (55.9)	5251 (40.1)	4199 (31.9)	
Race					<.001
Mexican American	2933 (22.4)	2701 (20.6)	2349 (17.9)	1801 (13.7)	
Other Hispanic	1277 (9.7)	1125 (8.6)	1056 (8.1)	831 (6.3)	
Non-Hispanic White	5471 (41.7)	5615 (42.8)	5781 (44.1)	5948 (45.1)	
Non-Hispanic Black	2270 (17.3)	2479 (18.9)	2680 (20.5)	3438 (26.1)	
Other Race	1164 (8.9)	1210 (9.2)	1236 (9.4)	1162 (8.8)	
Education level					<.001
<High school	3561 (27.2)	3773 (28.7)	3664 (28.0)	3742 (28.4)	
High school graduate or general equivalency diploma	2912 (22.2)	3085 (23.5)	3209 (24.5)	3312 (25.1)	
>High school	6625 (50.5)	6258 (47.7)	6212 (47.4)	6105 (46.3)	
Unknown	17 (0.1)	14 (0.1)	17 (0.1)	21 (0.2)	
Ratio of family income to poverty					<0.001
≤1	2905 (22.2)	2649 (20.2)	2575 (19.7)	2426 (18.4)	
1–3	4759 (36.3)	5026 (38.3)	4964 (37.9)	5326 (40.4)	
>3	4252 (32.4)	4323 (32.9)	4462 (34.1)	4283 (32.5)	
Unknown	1199 (9.1)	1132 (8.6)	1101 (8.4)	1145 (8.7)	
BMI					<.001
<30 kg/m^2^	9551 (72.8)	8133 (61.9)	7001 (53.4)	5437 (41.3)	
≧30 kg/m^2^	2211 (16.9)	3564 (27.1)	4666 (35.6)	6156 (46.7)	
Unknown	1353 (10.3)	1433 (10.9)	1435 (11.0)	1587 (12.0)	
Smoking					<.001
No	8519 (65.0)	7811 (59.5)	7223 (55.1)	6544 (49.7)	
Yes	4596 (35.0)	5319 (40.5)	5879 (44.9)	6636 (50.3)	
Alcohol use					<.001
No	6175 (47.1)	5565 (42.4)	5033 (38.4)	4905 (37.2)	
Yes	6940 (52.9)	7565 (57.6)	8069 (61.6)	8275 (62.8)	
Hypertension					<.001
No	10,475 (79.9)	9590 (73.0)	8690 (66.3)	6702 (50.8)	
Yes	2640 (20.1)	3540 (27.0)	4412 (33.7)	6478 (49.2)	
Hypercholesterolemia					<.001
No	10,209 (77.8)	9586 (73.0)	9051 (69.1)	8402 (63.7)	
Yes	2906 (22.2)	3544 (27.0)	4051 (30.9)	4778 (36.3)	
Diabetes					<.001
No	12,018 (91.6)	11,884 (90.5)	11,682 (89.2)	11,025 (83.6)	
Yes	1097 (8.4)	1246 (9.5)	1420 (10.8)	2155 (16.4)	
Heart disease					<.001
No	12,569 (95.8)	12,325 (93.9)	12,057 (92.0)	11,278 (85.6)	
Yes	546 (4.2)	805 (6.1)	1045 (8.0)	1902 (14.4)	
Stroke					<.001
No	12,834 (97.9)	12,764 (97.2)	12,662 (96.6)	12,427 (94.3)	
Yes	281 (2.1)	366 (2.8)	440 (3.4)	753 (5.7)	
ALT, U/L	20.0 (15.0–37.0)	23.0 (16.0–44.0)	25.0 (18.0–47.0)	26.0 (18.0–47.0)	<.001
AST, U/L	21.0 (18.0–25.0)	22.0 (19.0–26.0)	23.0 (20.0–28.0)	24.0 (20.0–29.0)	<.001
Total protein, g/dL	7.2 ± 0.5	7.2 ± 0.5	7.2 ± 0.5	7.2 ± 0.5	.008
Globulin, g/dL	2.9 ± 0.4	2.9 ± 0.4	3.0 ± 0.4	3.1 ± 0.5	<.001
Creatinine, μmol/L	61.9 (53.0–71.6)	70.7 (61.9–81.3)	79.6 (66.3–88.4)	87.5 (72.5–102.5)	<.001
Uric acid, mg/dL	3.8 ± 0.6	4.9 ± 0.5	5.8 ± 0.6	7.2 ± 1.1	<.001
Albumin, g/dL	4.3 ± 0.3	4.3 ± 0.4	4.2 ± 0.4	4.1 ± 0.4	<.001

ALT = alanine aminotransferase, AST = aspartate aminotransferase, BMI = body mass index, SD = standard deviation.

### 3.2. Association between UAR and all-cause mortality

**Figure 2.**
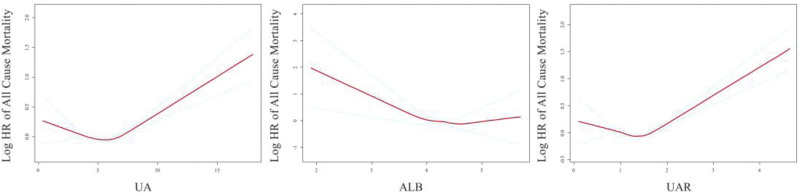
Smoothed curves fitting. Adjusted for age, race, education level, ratio of family income to poverty, BMI, smoking, alcohol use, ALT, AST, total protein, globulin, creatinine, and histories of hypertension, hypercholesterolemia, diabetes, heart disease, and stroke. ALT = alanine aminotransferase, AST = aspartate aminotransferase, BMI = body mass index.

**Figure 3.**
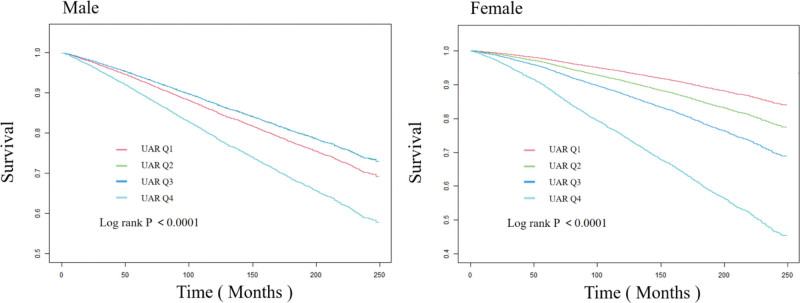
K-M curves. K-M = Kaplan–Meier.

**Figure 4.**
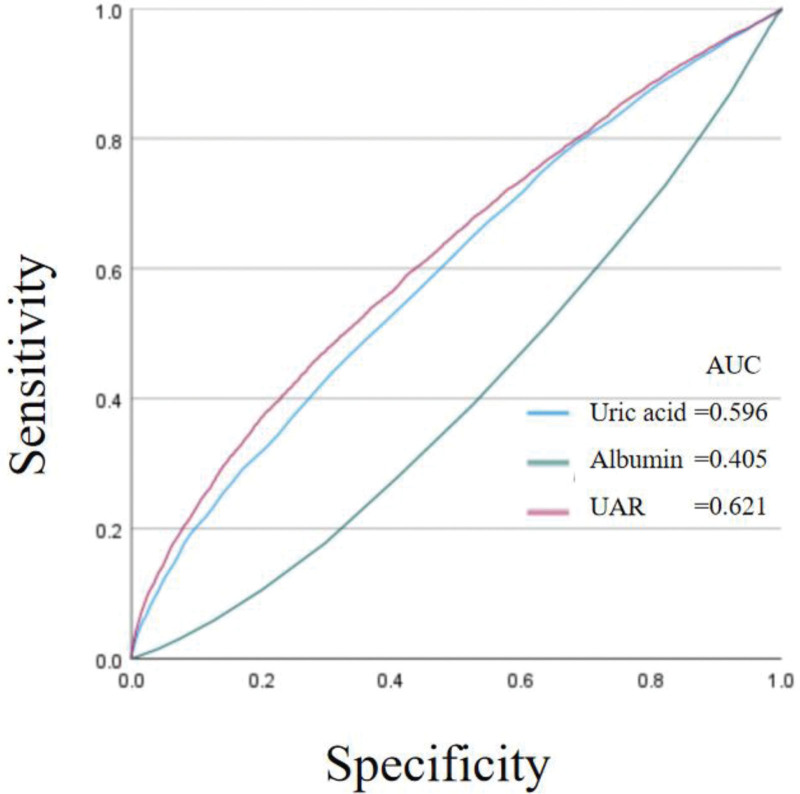
ROC curves. ROC = receiver operating characteristic.

Smooth curves fitting was employed to investigate the association between UA, ALB, UAR, and all-cause mortality, revealing a U-shaped relationship with a distinct inflection point (Fig. 2). Subsequently, threshold saturation effect analysis was conducted to assess the statistical significance of the segmented regression correlation (Table 2). With the increase of UAR, the mortality risk of participants initially decreased and then increased. There was a noticeable inflection point, with the inflection point level for females being lower than that for males. For male participants, when UAR < 1.51, the risk of death gradually decreased as UAR increased (HR = 0.685, 95% CI: 0.568–0.825, *P* < .0001). When UAR > 1.51, the mortality risk increased with rising UAR (HR = 1.787, 95% CI: 1.534–2.082, *P* < .0001). For female participants, when UAR < 1.34, the risk of death gradually decreased as UAR increased (HR = 0.782, 95% CI: 0.619–0.988, *P* < .0001). When UAR > 1.34, the mortality risk increased with rising UAR (HR = 1.494, 95% CI: 1.286–1.735, *P* < .0001). The KM curves suggested that the high UAR group (1.49 < UAR < 4.65) had a lower survival rate than the low UAR group (0 < UAR < 1.03) for both female and male participants, the Log Rank *P* < .0001 (Fig. 3). The result of receiver operating characteristic curves indicates that compared with uric acid and albumin, UAR has a better prediction effect on all-cause mortality (Fig. 4).

**Table 2:** Threshold saturation effect analysis.

	Uric acid	Albumin	UAR
	HR (95% CI) *P*	HR (95% CI) *P*	HR (95% CI) *P*
	K_uric acid_ = 6.50	K_albumin_ = 4.00	K_UAR_ = 1.51
Male			
< K	0.920 (0.881–0.961) .0002	0.437 (0.241–0.790) .0062	0.685 (0.568–0.825) < .0001
> K	1.169 (1.118–1.224) < .0001	0.841 (0.471–1.503) .5586	1.787 (1.534–2.082) < .0001
Log-likelihood ratio test	<0.0001	<0.0001	<0.0001
	K_uric acid_ = 5.00	K_albumin_ = 4.10	K_UAR_ = 1.34
Female			
< K	0.917 (0.853–0.986) .0190	0.149 (0.027–0.818) .0285	0.782 (0.619–0.988) .0395
> K	1.088 (1.049–1.128) < .0001	0.358 (0.064–1.990) .2404	1.494 (1.286–1.735) < .0001
Log-likelihood ratio test	<0.0001	<0.0001	<0.0001

Adjusted for age, race, education level, ratio of family income to poverty, BMI, smoking, alcohol use, ALT, AST, total protein, globulin, creatinine, and histories of hypertension, hypercholesterolemia, diabetes, heart disease, and stroke.

ALT = alanine aminotransferase, AST = aspartate aminotransferase, BMI = body mass index, UAR = uric acid to albumin ratio.

### 3.3. Effect of UAR on death from heart disease

**Figure 5.**
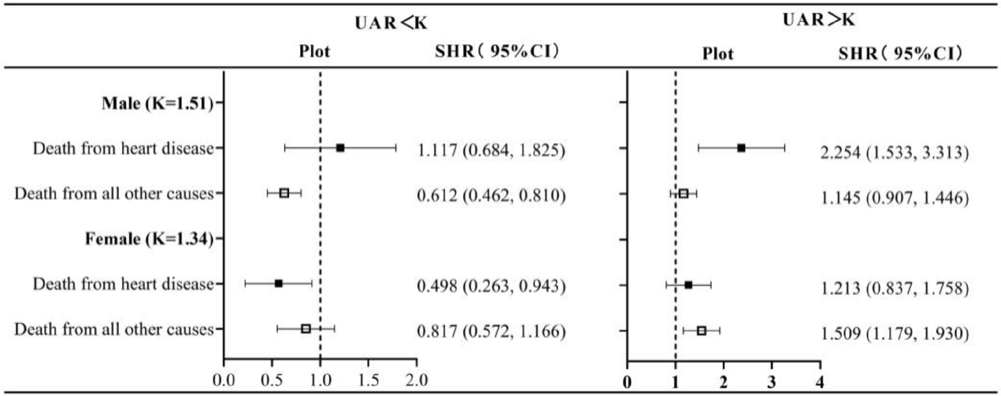
Effect of UAR on death from heart disease. Adjusted for age, race, education level, ratio of family income to poverty, BMI, smoking, alcohol use, ALT, AST, total protein, globulin, creatinine, and histories of hypertension, hypercholesterolemia, diabetes, heart disease, and stroke. ALT = alanine aminotransferase, AST = aspartate aminotransferase, BMI = body mass index.

The effect of UAR on heart disease death was assessed using competing risk models. The results suggested that for male patients in group of UAR > 1.51, the risk of death from heart disease increases with UAR (SHR = 2.254, 95% CI: 1.533–3.313; Fig. 5).

## 4. Discussion

To our knowledge, this study represents the first analysis of the association between UAR and all-cause mortality in United States adults. Our findings demonstrate that an elevated UAR is associated with more severe disease status and a higher burden of complications. Furthermore, UAR not only shows a significant association with but also acts as an independent predictor of all-cause mortality in this population, following a U-shaped relationship. Notably, UAR exhibited superior predictive performance for all-cause mortality compared to using urea or albumin alone (ROC_UAR_ = 0.621). These findings underscore the potential clinical utility of the UAR as a novel and robust biomarker for risk stratification and the early identification of high-risk individuals susceptible to mortality, thereby offering valuable prognostic insight for clinical decision-making.

Our study found that the high UAR group (1.49 < UAR < 4.65) had a lower survival rate than the low UAR group (0 < UAR < 1.03; 22.9% vs 9.4%, *P* < .001). The high UAR population was characterized by elder, smoking, drinking, hypertension, hyperlipidemia, cardiac death, diabetes, and stroke, alongside higher creatinine, uric acid, and globulin levels, and lower albumin levels. Some studies have indicated that an elevated UAR is significantly associated with CAD severity in patients with NSTEMI (OR = 2.08; 95% CI: 1.21–3.58),^[12]^ and in STEMI, a higher UAR was independently predictive of increased mortality (HR = 1.33, 95% CI: 1.16–1.52).^[13]^ In patients with acute kidney injury, an elevated admission UAR ≥ 2.4 mg/g independently predicts 30-day mortality, the high-UAR patients had a markedly reduced survival rate (63.7% vs 85.9%, *P* = .001).^[17]^ Notably, UAR was identified as an independent predictor of long-term cardiac mortality in patients with unstable angina pectoris after percutaneous coronary intervention, and multivariate Cox regression analysis confirmed that an elevated UAR was independently associated with increased long-term cardiac mortality (HR = 1.26, 95% CI: 1.08–1.46, *P* = .004).^[18]^ These findings, consistent with the results of this study, suggests that UAR levels can serve as a new indicator to assess the severity of patients.

Our analysis revealed a nonlinear U-shaped association between UAR levels and all-cause mortality in the adult population, as opposed to a linear correlation. As UAR levels increased, the risk of death initially decreased and then rose, with a noticeable inflection point. It is worth noting that the views of men and women are not consistent. The inflection point for women was lower than that for men; specifically, the inflection point for men was at 1.51, while for women, it was at 1.34. Large-scale health examination data have established that the reference interval for serum uric acid is consistently higher in males than in females across comparable age groups.^[19]^ Furthermore, a clinical study indicated that among patients with HF, females exhibit significantly lower serum uric acid levels compared to males (5.9 mg/dL vs 6.6 mg/dL).^[20]^ Our study similarly revealed a nonlinear relationship between serum uric acid levels and all-cause mortality, with a notably earlier inflection point observed in females compared to males. Consistent with this, a cohort study has established that men exhibit mean serum albumin concentrations approximately 2% higher than those observed in women.^[21]^ UAR is calculated by dividing uric acid concentration by albumin concentration. As a derivative index influenced by both constituent variables, UAR levels consequently exhibit significant variation between sexes.

Our study identified serum uric acid as an independent risk factor for all-cause mortality. A U-shaped relationship between uric acid levels and mortality was identified in both Korean and American adults.^[22,23]^ It has been shown that UA levels ≥ 8 mg/dL predicted all-cause and cardiovascular disease-related mortality (HR = 1.13; 95% CI: 1.06–1.21), particularly in the presence of malnutrition, especially in elderly individuals with malnutrition (HR = 1.19; 95% CI: 1.01–1.32).^[24]^ Similarly, studies involving patients with end-stage renal disease or individuals with normal baseline renal function have demonstrated that UA is an independent risk factor (HR = 1.13; 95% CI: 0.99–1.29). Furthermore, in patients with normal renal function, UA levels exceeding 8.0 mg/dL were associated with significantly elevated risks of developing chronic kidney disease within 2 years (RR = 2.91, 95% CI: 1.79–4.75 in men; RR = 10.39, 95% CI: 1.91–56.62 in women).^[25,26]^

A significant inverse association was observed between albumin concentrations and all-cause mortality in our study population. Patients with severe forms of myocardial infarction or injury, HF, stroke, hip fracture, malignancy, and renal disease often exhibit decreased levels of serum albumin (hypoalbuminemia),^[27,28]^ which serves as a robust, reliable, and independent prognostic marker in these populations. Other studies revealed a strong inverse association between serum albumin levels and mortality (HR = 191; 95% CI: 1.82–2.01), underscoring the predictive value of serum albumin and confirming that low albumin levels are associated with increased short- and long-term mortality.^[21,28]^ In the Copenhagen General Population Study, a nearly linear association between albumin levels and CVD incidence was observed during a median follow-up of 8.5 years, and each 10 g/L decrease in albumin was significantly associated with an increased incidence of CVD (HR = 1.96; 95% CI: 1.43–2.68).^[9]^ Similarly, a meta-analysis demonstrated a strong association between hypoalbuminemia and adverse clinical outcomes, while each 10 g/L decline in serum albumin concentration significantly increased the odds of mortality by 137%, morbidity by 89%, prolonged intensive care unit and hospital stays by 28% and 71%, respectively (OR = 2.37; 95% CI 2.10–2.68), and this association between hypoalbuminemia and poor outcomes appeared to be independent of both nutritional status and inflammation.^[29]^ Our study findings align with these previous investigations.

As previously noted, several studies indicated that low serum albumin levels and high uric acid levels are both associated with an elevated risk of all-cause mortality.^[30,31]^ Given these considerations, relying solely on individual indicators such as uric acid or albumin levels may not always provide accurate predictions of all-cause mortality. Therefore, there is a need to identify more comprehensive indicators for predicting all-cause mortality in populations. UAR combines both uric acid and serum albumin levels in the body and serves as a recently introduced inflammatory marker reflecting inflammation, oxidative stress, and proteolysis; notably, UAR has demonstrated a higher predictive value than uric acid or albumin alone.^[32]^

Our study revealed that higher UAR levels were predictive of increased all-cause mortality, independently of other predictors such as uric acid and albumin levels, nutritional status, age, and BMI, thereby indicating its reliability (HR = 1.494, receiver operating characteristic_UAR_ = 0.621). In recent years, UAR has emerged as a potential predictor of events related to cardiovascular disease. A study involving 4599 patients with STEMI treated with PCI revealed that elevated UAR was associated with increased long-term mortality in these patients (increase from 1.15 to 1.73; HR 1.33), independent of other classical risk factors; and the authors concluded that UAR serves as a prognostic indicator of mortality in STEMI patients and can readily identify high-risk individuals.^[13]^ Another study investigated the relationship between UAR and disease severity in patients with chronic CAD using the syntax score as an indicator of CAD severity. The study found that UAR levels were significantly elevated in patients with syntax score > 22. In a multivariate analysis model, UAR was identified as a superior predictor of disease severity, with a likelihood ratio of 60.95 (OR = 2.08; 95% CI 1.21–3.58; *P* = .008). Thus, UAR can serve as an objective and reliable inflammatory marker for predicting the degree of CAD in patients with NSTEMI.^[12]^ And elevated UAR may be an independent and effective biomarker for predicting poorly developed coronary collateral circulation development in NSTEMI patients.^[33]^ Besides, a study investigated whether preoperative UAR could predict lymph node metastasis in patients with non-small cell lung cancer undergoing VATS. The findings indicated an association between lymph node metastasis and UAR (*P* = .03; OR: 2.6), suggesting that preoperative UAR can predict lymph node metastasis in non-small cell lung cancer patients undergoing VATS surgery.^[34]^ The association between elevated uric acid and inflammatory processes/oxidative risk, as well as the link between low serum albumin and heightened inflammatory and catabolic processes, may elucidate the ability of UAR to predict the risk of all-cause mortality in patients.^[35]^ The results of previous studies are consistent with ours, UAR can be regarded as an easily accessible parameter for predicting all-cause mortality in patients.

## 5. Limitations

This study has several limitations. Missing data for certain confounding factors were addressed via mean imputation, which may introduce minor bias; however, as these missing values accounted for <1% of observations, the results remain methodologically robust. The NHANES database provides only single-timepoint measurements of UAR levels, which may not represent long-term exposure averages. Due to the inherent limitations of observational research, we cannot establish causal relationships. Although we made diligent attempts to include for all potential confounding variables, it is plausible that unidentified or unmeasured factors could have influenced our findings.

## 6. Conclusion

In conclusion, UAR demonstrates utility as an accessible and potentially valuable novel inflammatory biomarker that may aid in identifying patients at elevated mortality risk. However, its validation as a robust independent prognostic indicator requires confirmation through prospective studies accounting for residual confounding.

## Acknowledgments

We would like to thank MedSci for English language editing (https://www.medsci.cn/).

## Author contributions

**Data curation:** Jie Li, Liang Chen.

**Formal analysis:** Mengmeng Shan, Liang Chen, Lili Wang.

**Funding acquisition:** Mengmeng Shan, Jinzhou Zhu.

**Investigation:** Suling Ye, Mengmeng Shan, Jinzhou Zhu.

**Methodology:** Jie Li, Jinzhou Zhu, Liang Chen.

**Software:** Jie Li, Liang Chen.

**Supervision:** Jinzhou Zhu, Liang Chen, Lili Wang.

**Validation:** Jie Li, Jinzhou Zhu, Liang Chen, Lili Wang.

**Visualization:** Suling Ye, Jie Li, Lili Wang.

**Writing – original draft:** Suling Ye, Jie Li.

**Writing – review & editing:** Mengmeng Shan, Jinzhou Zhu, Liang Chen, Lili Wang.
